# Relationship between cognitive processing, language and verbal fluency among elderly individuals

**DOI:** 10.1590/1980-57642018dn13-030006

**Published:** 2019

**Authors:** Helen Capeleto Francisco, Allan Gustavo Brigola, Ana Carolina Ottaviani, Ariene Angelini dos Santos-Orlandi, Fabiana de Souza Orlandi, Francisco José Fraga, Letícia Pimenta Costa Guarisco, Marisa Silvana Zazzetta, Renata Valle Pedroso, Sofia Cristina Iost Pavarini

**Affiliations:** 1 Federal University of São Carlos Graduate Program in Nursing São CarlosSP Brazil Federal University of São Carlos (UFSCar), Graduate Program in Nursing, São Carlos, SP, Brazil.; 2 Federal University of São Carlos Graduate Program in Gerontology São CarlosSP Brazil Federal University of São Carlos (UFSCar), Graduate Program in Gerontology, São Carlos, SP, Brazil.; 3 Federal University of ABC Engineering, Modelling and Applied Social Sciences Center Santo AndréSP Brazil Federal University of ABC (UFABC), Engineering, Modelling and Applied Social Sciences Center (CECS), Santo André, SP, Brazil.

**Keywords:** elder, aging, event-related potential, P300, language, idoso, envelhecimento, potencial relacionado ao evento, P300, linguagem

## Abstract

Some cognitive dimensions, such as attention, memory and executive functions, may decline with age, while other functions remain intact or even improve due to greater life experience.

**Objective:**

to analyze the relationship between cognitive processing, language and verbal fluency among elderly individuals seen by primary healthcare services located in a city in the interior of São Paulo, Brazil.

**Methods:**

a cross-sectional study with a quantitative method was conducted. A total of 149 elderly individuals were assessed through previously scheduled interviews. Data collection included a questionnaire on sociodemographic data and the Addenbrooke's Cognitive Examination - Revised (ACE-R). Cognitive processing (P300) was assessed using a device that captures potentials elicited in auditory tasks. Descriptive analysis and Spearman's correlation were performed with the level of significance established at 5%.

**Results:**

a negative correlation was found between language and P300 latency, while a positive correlation was found between verbal fluency and P300 amplitude. Comprehension and naming tasks showed a negative correlation with latency. The repetition task revealed a positive correlation with P300 amplitude.

**Conclusion:**

although more extensive testing is needed, these findings suggest that language correlates with P300 latency, whereas verbal fluency correlates with P300 amplitude.

## RESUMO

As dimensões cognitivas, como atenção, memória e funções executivas, podem diminuir com a idade, enquanto outras funções permanecem ou até melhoram devido a uma maior experiência de vida.

**Objetivo:**

analisar a relação entre processamento cognitivo, linguagem e fluência verbal em idosos atendidos em serviços de atenção básica localizados em um município do interior de São Paulo, Brasil.

**Métodos:**

estudo transversal, método quantitativo. Um total de 149 idosos foram avaliados por meio de entrevistas previamente agendadas. A coleta de dados incluiu um questionário abordando dados sociodemográficos e o Exame Cognitivo de Addenbrooke - Revisado (ACE-R). O processamento cognitivo (P300) foi avaliado por meio de um dispositivo que capta potenciais elicitados em tarefas auditivas. A análise descritiva e a correlação de Spearman foram realizadas com o nível de significância estabelecido em 5%.\

**Resultados:**

foi encontrada correlação negativa entre a linguagem e a latência do P300 e uma correlação positiva entre a fluência verbal e a amplitude do P300. Tarefas de compreensão e nomeação apresentaram uma correlação negativa com a latência. A tarefa de repetição revelou correlação positiva com a amplitude do P300.

**Conclusão:**

embora testes mais extensos sejam necessários, esses achados sugerem que a correlação de linguagem com a latência e fluência do P300 está correlacionada com a amplitude do P300.

Some cognitive dimensions, such as attention, memory and executive functions, may decline with age, while other functions remain intact or even improve due to greater life experience.^1^^-^3 In the case of language, decline is not homogenous because some aspects may be preserved, such as articulation of words, synthetic processing and vocabulary, while pragmatic and discursive skills, word-naming and verbal fluency are the aspects most frequently compromised by the aging process due to the deterioration of basic cognitive functions, i.e., memory and executive functions.^4^^,^^5^

There is evidence that cognitive processing declines with aging,^6^ which would explain the difficulty in performing tasks that require speed, concentration, and inductive reasoning, while tasks such as reading, understanding or memorizing, take longer.^7^ It is however, necessary to clearly understand how the brain of a healthy elderly individual works in order to identify when symptoms such as slowness, difficulties performing simultaneous tasks or recalling recent facts indicate pathological cognitive changes.^8^^-^11

Cognitive assessment requires the measurement of primary cognitive domains such as memory, language, executive functions and visuospatial skills.^12^ Various instruments have been created in order to assess these skills quickly and reliably.^12^ The Addenbrooke's Cognitive Examination was developed in 2000^13^ and revised in 2006 (ACE-R)^14^ to assess cognitive skills. The ACE-R was translated into Portuguese and adapted for the Brazilian culture in 2007,^15^ and its interpretation depends on educational level.^12^^,^^16^^,^^17^

An electrophysiological, or cognitive processing, test (P300) was used to complement the behavioral cognitive assessment.^18^^,^^19^ The P300 wave is an event-related potential component that, when recorded by electroencephalography, surfaces as a positive deflection in voltage with a latency of approximately 300 ms after an auditory or visual stimulus is presented, the bioelectrical responses of which are related to attention, memory and decision-making.^18^^,^^19^ The P300 is mainly elicited using the oddball paradigm, in which participants are instructed to detect low-probability target items that occur randomly within a sequence of high-probability non-target items.^20^

Because the P300 is elicited by stimuli that require a high level of cognitive processing, it is considered one of the most promising electrophysiological tests for assessing dysfunctions and/or changes in the central nervous system and may be useful for early diagnosis of pathological cognitive changes. However, although P300 may be a promising instrument in terms of research, it has not yet gained wide acceptance in clinical or neuropsychological practice, because there is high variability in the values of latency and amplitude, making it impractical to use this technique for clinical and diagnostic purposes.^20^^-^22

This study was prompted by the question: Are P300 results related to results obtained by the ACE-R on the language and verbal fluency domains? For this reason, this study's objective was to analyze the relationship between cognitive processing, language and verbal fluency of elderly individuals seen by primary healthcare services located in a city in the state of São Paulo, Brazil.

## METHODS

This cross-sectional study is based on the assumptions of quantitative research and is part of a larger study called “Primary Health Care Follow-up of Elderly Caregivers” that was conducted by the Health and Aging Research Group at the Federal University of São Carlos (UFSCar), that collected sociodemographic, health, cognitive, psychological and functional data of elderly people registered at the Family Health Units of a city in the interior of São Paulo.

The sample was randomly recruited from individuals living in the area covered by the Family Health Units. The sample was calculated with the significance level, or alpha, established at 5% (type1 error) and power of 80% (beta or type 2 error at 20%). Mean and standard deviation were based on a previous study of elderly individuals; a sample of 149 individuals was achieved.

The following inclusion criteria were adopted: being aged 60 years or older and being registered with one of the Health Family Units located in the city. Individuals with cognitive or language impairment that impeded an interview, severe hearing impairment, a history of stroke with severe sequelae, use of alcohol or psychoactive drugs that hindered understanding and ability to answer the instruments, were excluded. Other individuals were also excluded either because the researchers were unable to contact them via telephone or at home after three attempts made on alternative days and at different times, had died, changed address, or refused to participate.

This study complied with ethical guidelines of Resolution No. 466/2012^23^ and was approved by the Institutional Review Board at the Federal University of São Carlos (CAAE: 80458017.7.0000.5504). All the participants provided a written free and informed consent form.

The interviews and assessments were conducted from June 2016 to July 2017. Data were collected in two stages. In the first stage, the interviewers visited all the elderly individuals listed by the health services in order to verify whether they met inclusion or exclusion criteria. Those who met the inclusion criteria were invited to complete the interview and, after their consent, a questionnaire collecting sociodemographic information, such as age, sex, education, marital status, family income, ethnicity, whether the individual lived alone and health information was collected. A time was then scheduled for the second part of the interview, with a maximum interval of one week between the two stages.

Data on cognitive processing and cognition were collected at the second stage. In order to ensure that a calm, silent and well-lit room would be available, we previously arranged a place easily accessible for the participants, located in the neighborhood.

Addenbrooke's Cognitive Examination - Revised (ACE-R) was applied to all the individuals in the sample. This short cognitive assessment battery is useful to detect dementia, differentiating Alzheimer's disease from frontotemporal dementia in its early stages, and to diagnose mild cognitive impairment in the elderly population in general. This questionnaire evaluates cognitive domains: orientation and attention (18 points), memory (26 points), verbal fluency (14 points), language (26 points) and visuospatial skills (16 points), with a score that ranges from 0 to 100 points. The tasks of the Mini-Mental State Examination (MMSE) are part of this assessment and can be calculated separately. Language is assessed through tasks focused on comprehension, reading, writing, repetition and naming figures, with a cut-off point of 22. Verbal fluency is divided by semantic category (naming animals) and phonemic category (words initiated with letter P) with a cut-off point of eight.^12^^,^^15^^,^^16^

A Neuron-Spectrum-4/EPM device, from Neurosof, was used to perform the electrophysiological evaluation of cognitive processing (P300). The participants were accommodated on a comfortable chair placed in a quiet room, properly equipped for the exam. The electrodes were affixed according to the 10/20 system over the frontal region (Fz), vertex (Cz) and parietal region (Pz). Reference electrodes were placed on the right (A1) and left (A2) ear lobes, interconnected by cables. The electrodes were connected to the preamplifier and impedance was kept below 5 KΩ.

An auditory stimuli sequence was presented in a binaural fashion using Ear-Tone 3A ear bud headphones containing two signs of the same intensity (90dBNA). Within this sequence, frequent or standard stimulus (1000Hz) was triggered 80% of the time, while the low-frequency stimulus (2000 Hz) was randomly placed among the frequent stimuli 20% of the time. A total of 300 stimuli were given and each stimulus lasted 100 ms.

Note that potential peripheral auditory loss does not interfere in the P300 registry as long as the individual is able to hear the instructions and stimuli.^10^^,^^18^ In this study, the test was performed in such a way that stimuli were audible and comfortable for the participants.

The results generated by the electroencephalogram waves were stored on a computer and later sent for analysis to the Engineering, Modeling and Applied Social Sciences Center at the Federal University of ABC at São Bernardo do Campo, SP, Brazil according to the protocol provided by the Health and Aging Research Group.

Data were entered into an Excel for Windows spreadsheet and then analyzed on the Statistical Package for Social Sciences (SPSS), version 21.0. Descriptive statistics, including location and dispersion measures (mean, standard deviation, minimum and maximum, median) were used to describe continuous variables, while frequencies, with absolute values (n) and percentages (%), were used to describe categorical variables. Given the non-normality of the variables, according to the Kolmogorov-Smirnov test, Spearman's correlation non-parametric test was used. The level of significance was established at 5% (p≤0.05). The magnitude of correlations was classified into: weak <0.29; moderate 0.3-0.59; strong 0.6-0.9 and perfect =1.0.^24^

## RESULTS

The sample was composed of 149 elderly individuals with mean age of 70.31 (±6.75). Of the total participants, 118 were women (79.2%) and 31 men (20.8%). Average education was 3.76 years (±3.19). Health characteristics were self-reported: 87.82% (131) reported hypertension, 20.13% (30) were smokers, and 3.36% (5) alcoholics. The performance of 51 (34.22%) elderly individuals attained the cut-off point on the ACE-R expected for educational level, while 98 elderly individuals (65.78%) scored below the cut-off point on the ACE-R, i.e. lower than expected score for level of education. The cut-off scores adopted were 83 points for individuals with ≥5 years of education and 65 points for individuals with <5 years of education.^12^

Table 1 presents the correlation between P300 latency and amplitude and the performance of elderly individuals on cognitive domains assessed by the ACE-R.

**Table 1:** Correlation matrix between P300 latency and amplitude and performance of elderly individuals on cognitive domains assessed by the ACE-R (n=149). São Carlos, SP, Brazil, 2017.

		P300 Latency				P300 Amplitude		
		Fz	Cz	Pz		Fz	Cz	Pz
	**n**	**116**	**133**	**139**		**116**	**133**	**139**
Total ACE-R	r	-0.22	-0.15	-0.10		0.24	0.23	0.22
	p-value	0.02	0.08	0.24		0.01	0.01	0.01
Language	r	-0.27	-0.19	-0.13		0.12	0.18	0.17
	p-value	<0.001	0.02	0.12		0.03	0.04	0.05
Verbal fluency	r	-0.17	-0.06	-0.07		0.28	0.19	0.19
	p-value	0.07	0.51	0.45		<0.001	0.02	0.02
Attention and orientation	r	-0.13	-0.04	-0.02		0.22	0.26	0.21
	p-value	0.17	0.62	0.77		0.03	<0.001	0.01
Memory	r	-0.13	-0.13	-0.07		0.19	0.22	0.23
	p-value	0.16	0.15	0.41		0.04	0.01	0.01
Visuospatial ability	r	-0.23	-0.16	-0.13		0.16	0.13	0.10
	p-value	0.01	0.07	0.12		0.09	0.14	0.24

Spearman's correlation test.

The correlation between P300 latency and amplitude and performance of elderly individuals on language tasks assessed by the ACE-R is presented in Table 2.

**Table 2:** Correlation matrix between P300 latency and amplitude and performance of elderly individuals on language tasks assessed by the ACE-R (n=149). São Carlos, SP, Brazil, 2017.

		P300 Latency				P300 Amplitude		
		Fz	Cz	Pz		Fz	Cz	Pz
	n	**116**	**133**	**139**		**116**	**133**	**139**
Language - Comprehension	r	-0.27	-0.14	-0.11		0.19	0.13	0.14
	p-value	<0.001	0.11	0.22		0.04	0.15	0.11
Language - Repetition	r	-0.12	-0.13	-0.01		0.13	0.22	0.12
	p-value	0.21	0.14	0.24		0.18	0.01	0.17
Language - Naming	r	-0.25	-0.19	-0.11		0.19	0.15	0.17
	p-value	0.01	0.03	0.21		0.05	0.01	0.05
Language - Reading and writing	r	-0.13	-0.16	-0.15		0.13	0.13	0.05
	p-value	0.16	0.07	0.09		0.15	0.15	0.54

Spearman's correlation test.

## DISCUSSION

The P300 test measures responses generated by cortical electrophysiological potentials using cognitive tasks that require attention and working memory. Two variables are used to assess P300: amplitude and latency. Latency refers to the temporal processing of information and amplitude is a variable related to attention level. There is evidence that cognitive processing changes with age, leading to increased latency and decreased amplitude.^6^^,^^11^

Because language and verbal fluency are cognitive skills directly dependent on attention and working memory, this study hypothesis was that there is a correlation between cognitive potential - P300 and language and verbal fluency. This hypothesis is supported by authors^25^ who believe that the production of language involves planning and monitoring. As such, language production is not a totally automatic task, because it requires cognitive processing. Statistically significant correlations, in the expected direction, were consistently found between the P300 and performance on verbal fluency and language tests; however, correlations were weak, which partially contradicts the study's initial hypothesis. Thus, only the more robust correlations in language and verbal fluency are discussed here, that it, only statistically significant results (p<0.05) with r >0.20.

In relation to amplitude, the study results showed positive correlations between the P300 wave and verbal fluency on channel Fz and the repetition task on channel Cz. When P300 wave latency was assessed, the results indicated negative statistically significant correlations with total language, as well as with comprehension and naming tasks on channel Fz, showing that, as language performance improves, latency of the P300 wave decreases. Note that no statistically significant correlations were found between verbal fluency and P300 latency.

The sites where P300 is generated via auditory stimuli involve structures of the frontal cortex, supratemporal auditory cortex and hippocampus,^10^^,^^18^^,^^19^ which are brain areas directly related to executive functions and language, which could explain the correlations between the results of the tests analyzed here, especially the role of channel Fz. A previous study analyzing a P300 age-related “anterior displacement” pattern found posterior responses in young individuals and predominantly frontal responses among elderly individuals. An increased use of frontal executive functions would result in an increase in the wave amplitude as a compensatory mechanism to perform the P300 tasks properly.^26^

The verbal fluency test depends on the proper functioning of working memory. A study^27^ found that P300 amplitude in adult and elderly individuals varied with the efficiency of working memory. Advanced P300 activity was found at the frontal electrode of participants while working memory was being used, unlike younger individuals, in whom greater posterior activation was found. Another study of individuals with posttraumatic brain injury^28^ found that worse performance on neuropsychological tests was associated with decreased P300 amplitude relative to controls, although a typical P300 parietal wave response was evident. On the other hand, participants who performed better on the neuropsychological tests had robust amplitude for the P300, characterized by the recruitment of anterior brain regions, in addition to parietal activation. The recruitment of frontal areas in posttraumatic brain injury may represent compensatory neural mechanisms used to successfully maximize the performance of a task. The results of these studies may be corroborated by the present study findings, in as far as better performance on verbal fluency tasks, which assess executive functions and working memory, correlated with greater P300 amplitude on the Fz channel among elderly individuals. This result may indicate that the frontal region compensates for brain dysfunction accrued from aging.

A statistically significant correlation was found in this study between latency and the general score on the language - comprehension and naming tasks. These findings corroborate those of other studies indicating an increase in P300 latency during aging,^6^^,^^11^^,^^29^ which may suggest a worsening of attention and working memory and, consequently, of language.

Researchers^28^ hold that P300 may be a sensitive marker of neural plasticity that could be used to improve functional results in paradigms of cognitive remediation. In view of the correlations reported here, we assume that stimulation of cognition in the practice setting, especially attention and working memory, may result in improved verbal fluency and language among elderly individuals. Further studies, however, are needed to verify this assumption.

The studies investigating P300 among elderly individuals are heterogeneous with regard to the number of channels studied, sensory cues used (visual or auditory), method of application (pure tone, syllables, simple or complex phrases) and tasks involved (P300 alone or concomitantly with cognitive tasks). Recent studies^11^^,^^29^ have analyzed the Pa and Pb components of the P300 wave, seeking evidence of the Pb peak as a neurophysiological marker for pathological cognitive changes. No similar studies exploring the relationship between the P300 variables, verbal fluency and/or language among elderly individuals were found in the specialized literature. For this reason, the results of the present study cannot be compared directly with those of previous studies; studies on the P300 in elderly populations need to be consulted to strengthen this discussion. The lack of studies addressing this topic highlights the importance of further research to deepen this investigation.

The limitations of this study involve the method used to collect data, which was via a cognitive screening instrument, as opposed to a specific battery for assessing language composed of multiple tasks providing greater precision in the assessment of each language skill.^25^ The screening instrument was used because the present study was part of a larger investigation conducted in the homes of elderly individuals. Therefore, in order to better clarify these findings, future studies should compare the performance of elderly individuals on instruments assessing language and the P300, assessing pragmatic aspects and controlling for education. Future studies should also investigate P300 among individuals with dementia or conditions of a neurological nature that predominantly affect language, such as primary progressive aphasia (PPA), and studies exploring N400 and P600 in the elderly.

Although more extensive testing is needed, these findings suggest that language correlates with P300 latency, while verbal fluency correlates with P300 amplitude.
